# The current state of Spanish language resources for patients in Alabama with rare genetic disease: qualitative expert stakeholder interviews

**DOI:** 10.1007/s12687-026-00928-1

**Published:** 2026-07-31

**Authors:** Spencer Elizabeth Favor, Alicia Gomes, Carlos Javier Torres, Katie Church

**Affiliations:** 1 Department of Clinical and Diagnostic Sciences, Genetic Counseling Program, The University of Alabama at Birmingham, 1720 2nd Ave S, Birmingham, AL 35294 USA; 2 Hispanic and Immigrant Center of Alabama (¡HICA!), 117 Southcrest Drive, Birmingham, AL 35209 USA

**Keywords:** Spanish language, Rare disease, Patient resources, Qualitative, Genetic disorders

## Abstract

Background: Patients with a rare genetic disease face unmet needs deepened by the traditional American healthcare system. Social, educational, and financial support resources help address some of these challenges; however, for patients who are Spanish speaking, the language barrier makes it more difficult to attain such resources. This compounds with already existing healthcare disparities faced by the Latino/Hispanic community. This study explores the current state of resources available for patients in Alabama who are Spanish speaking with a rare genetic disease and identify specific areas for improvement. Methods: Eleven expert stakeholders who work with either rare genetic disease, patients who speak Spanish, or both, completed 1-hour online audio-only semi-structured interviews. Interviews focused on the current state of resources for this population and ideas for improvement. Transcripts were analyzed using reflexive thematic analysis. Results: A lack of sufficient access to Spanish speaking medical staff and interpreters prevents patients from accessing social support and the healthcare system. Additionally, digital or educational resources in Spanish are rarely accessible to patients. When resources are available they tend to be of high quality, but their implementation is inconsistent. This lack of accessible resources creates an undue burden for both patients and providers.

**Supplementary Information:**

The online version contains supplementary material available at https://doi.org/10.1007/s12687-026-00928-1.

## Significance

This study identified that the resources that currently exist are often high quality, however most patients and providers cannot easily access them. To improve quality of care and reduce provider burden, organizations need to focus on increasing Spanish language access through staff that speak Spanish and consistent, high-quality, Spanish language resource implementation.

**Supplementary Information:**

The online version contains supplementary material available at https://doi.org/10.1007/s12687-026-00928-1.

## Introduction

A rare disease is defined as a condition impacting less than 1 in 200,000 Americans (Rare Disorder Database 2024). While each of these conditions individually is rare, having a rare disease is common — affecting one in ten people (Genetic and Rare Diseases Information Center 2024). Although not all rare diseases are due to a single identifiable genetic cause, the scope of this research is specific to those with a genetic cause. It is estimated that between 72 and 80% of rare diseases have a genetic basis (Delgado-Vega et al. 2024). Therefore, the term “rare genetic disease” will be used. Patients with rare genetic diseases often have needs not met by the traditional healthcare system including facing high amounts of stigma, long diagnostic odysseys, significant travel to specialists, and providers unfamiliar with diagnosis and management of their condition (Belzer et al. 2022; Bryson and Bogart 2020). These factors contribute to higher levels of anxiety and depression, and lower health-related quality of life in those with rare genetic diseases compared to the general population (Bogart et al. 2022).

The term “resource” refers to sources of support, aid, or information that can assist patients with their health and quality of life. Resources include educational materials, financial aid, and what will be referred to as “human resources” ─ or the use of people and relationships as sources of socioemotional support. Educational resources have been shown to increase patient satisfaction, confidence, and compliance with medical advice and encourage people to take an active role in their care (Emiliana et al. 2019; Hansen et al. 2023; Pogue et al. 2018). Frequently, patients and caregivers turn to online search engines to locate educational resources about their condition (Litzkendorf et al. 2020). However, these searches are often unsuccessful, leaving patients and caregivers disappointed with the quality and quantity of information they hoped to find (Litzkendorf et al. 2020). Accurate, easily accessible resources are vital to combat misinformation or patients’ misinterpretation of information (Pogue et al. 2018). This is especially important in the rare genetic disease community as resources about these conditions are often scarce (Baumbusch et al. 2019; Litzkendorf et al. 2020).

A separate set of challenges impact individuals in the United States whose primary or native language is Spanish, referred to here as “Spanish speaking”. It is worth noting that “Hispanic” and “Latino” denote cultural identity and are sometimes used interchangeably in research. Since 70% of Latinos in the United States speak Spanish at home, as their primary language for daily communication and living, these cultural terms were used as a proxy for Spanish speaking individuals when reviewing previous literature (Lopez 2022). A study by Welty et al. showed 29.2% of patients who spoke Spanish reported their concerns were not fully addressed by providers, even with an interpreter present, compared to 10.2% of their English-speaking counterparts (Welty et al. 2012). Additionally, patients who spoke Spanish had to wait twice as long to see their healthcare professional (Welty et al. 2012). Hispanic/Latino patients with diabetes and other common conditions report communication issues with providers and not receiving adequate information about their health as barriers to quality healthcare (Amirehsani et al. 2017; Moreno et al. 2018). The concerns of Spanish speaking patients compound when individuals are affected by a rare genetic disease, rather than common conditions better addressed by traditional healthcare.

Patients who speak Spanish face barriers with accessing resources specifically for genetic conditions. Families that are Latino with children who have intellectual disabilities are less likely to participate in parental support programs or have access to healthcare services and educational interventions compared to non-Latino families (Cohen et al. 2014). One study found nearly two-thirds of Spanish speaking caregivers of individuals with Down syndrome, a genetic condition 285 times more common than a rare disease, felt frustrated and reported using “a lot of effort” to find Spanish language resources (Chung et al. 2023; Presson et al. 2013). Patient and caregiver listening sessions hosted by The National Organization for Rare Disorders found cost of care, lack of access to quality medical information, and difficulties with quality of life were some of the biggest issues facing Latinos with rare diseases (National Organization for Rare Disorders 2023). Although, some rare genetic disease organization have been working towards language inclusive websites and tools in recent years, there remains a deficit in quantity of Spanish language resources targeted to rare genetic diseases compared to English language resources (Tones et al. 2023; Westrate et al. 2020).

The lack of access to Spanish language resources is a problem that needs to be addressed as individuals who are Hispanic/Latino make up nearly one fifth of the United States population, with nearly 42 million of the country’s population speaking Spanish at home, making it the second most common spoken language in the United States (Sandy Dietrich 2022). The south is a region with a rapidly growing Spanish speaking population. In Alabama specifically, the Latino population increased 202% between 2000 and 2020 (Zong 2022). Overall, Alabama’s healthcare system performance is low, ranking 44th out of 50 (Radley et al. 2024). Specifically, the state’s Latino population faces significant health disparities compared to the White population, with healthcare system performance scores at the 8th and 61st percentiles respectively (Radley et al. 2024).

With care gaps for marginalized communities, including the Spanish speaking community, both the National Society of Genetic Counseling (NSGC) and the American College of Medical Genetics and Genomics (ACMG) acknowledge the need for equitable care and closing of current gaps (Confronting Racism, Oppression, & Inequity in Genetic & Genomic Medicine 2022; Matalon et al. 2023). However, these guidelines or statements focus on clinical practice and general healthcare access barriers. There are minimal policies or statements from United States-based genetics organizations targeted specifically to Spanish speaking communities and language resources. Therefore, this study evaluated the current landscape of Spanish language resources available to those with rare genetic diseases through semi-structured expert stakeholder interviews. Although the gap in resources is a national issue, this study was completed through a lens focused on the state of Alabama due to the research team’s location and the established low healthcare system performance for the Latino population.

## Methods

### Participants

This study was approved by the University of Alabama at Birmingham (UAB) Institutional Review Board (IRB-300012418). All participants had to be at least 18 years old and speak English. Participant eligibility was determined by self-selection of at least two of the following three statements: I engage with and/or my work impacts populations in Alabama, I engage with and/or my work impacts populations with rare genetic diseases, I engage with and/or my work impacts the Spanish-speaking population. Participants were then categorized into two groups. The first group was “Medical Professionals” including geneticists, genetic counselors, and non-genetics medical specialists. The second group was “Support Professionals” including social workers, language interpreters, and representatives from community support groups.

### Recruitment and procedure

Potential study participants were identified via web-search followed by a combination of direct recruitment and snowball sampling strategies. Snowball sampling was used due to the niche professional experiences necessary to meet inclusion criteria for this study and the small community of providers and support team members who are experts in rare diseases. An intake questionnaire (Supplemental Material 1) was sent via email to organizations and individuals between August and October 2024. The intake questionnaire included an informed consent document, multiple-choice questions about the individual’s professional background, and collection of contact information. The research team then contacted individuals who met inclusion criteria to schedule an interview.

### Interview design

The research team created a semi-structured interview guide (Supplemental Material 2) with questions tailored to each professional group through “problem-centered expert interview” techniques to explore professional knowledge and personal opinions (Doringer 2020). Interview questions were open-ended and asked about personal and professional background, experiences of their patients/clients, current resource use, and ideas for future resources. Participants were shown each question via PowerPoint during the interview to enhance comprehension, especially for those whose second language was English. A pilot interview was held with author CT, a member of the Hispanic/Latino community in Alabama, and edited based on feedback. Interviews were conducted in English by author SF via Zoom with audio only recording from September to November 2024. The recordings were de-identified and transcribed using Nvivo version 15. All references to specific institutions or individuals were replaced with a generic substitute.

### Coding and thematic analysis

The Standards for Reporting Qualitative Research (SRQR) checklist was used to ensure proper reporting (Supplemental Material 3) (O’Brien et al. 2014). Demographics of participants were analyzed with descriptive statistics. Two members of the research team (SF and KC) coded the data independently with inductive reflexive thematic analysis (Braun and Clarke 2019). This method was chosen because it allows for development of deep understanding and meaningful interpretation of participant experiences in relation to resource availability and quality (Braun and Clarke 2023). Both coders had a Bachelor of Arts in Spanish, cultural emersion experience (SF through study abroad in Spain and KC through dual-immersion primary school), healthcare experience working with the Spanish language community, and had lived in Alabama at least 3 years. This background mirrored the backgrounds of the medical professional group participants; therefore, the coders’ own experiences impacted their interpretation of the participant quotes.

The coders compared and discussed their inductive code applications to integrate both coders’ reflections and interpretations of the data and determine the final codebook. Each then independently applied the final codebook to each transcript. They then further discussed their second round of coding as a form of reflexive practice, combined their individual code applications, and assigned final codes to the data based on their shared interpretation. The coding team then constructed themes and sub-themes that best represented the data based on the final codes. Final codes, themes, and sub-themes were discussed and agreed upon by the entire research team consisting of four individuals of different ethnic, language, and professional backgrounds to give multiple lenses of interpretation and reflection.

## Results

### Participant characteristics

Sixty total recruitment emails were sent; sixteen responses were collected with fifteen individuals meeting inclusion criteria. Eleven individuals completed an interview (Table 1). Most participants (64%) identified as Hispanic/Latino, were fluent in Spanish, and engaged with the Hispanic/Latino culture both personally and professionally. The majority (82%) had patient interactions with both Spanish speaking individuals and those with rare genetic diseases. There were no participants whose patient interactions were only with individuals with rare genetic diseases. All participants were located in the United States at the time of their interview and ten out of 11 conducted all of their work in the United States. One participant also conducts work in Central and South America. She spoke broadly of her experiences, including both the United States and other Spanish-speaking countries. The de-identified job titles of each participant are listed in Table 2, with five medical professionals (designated as M#) and six support professionals (designated as S#). Although caregiver perspectives were not the goal of this research, one support professional (S4) was also a parent of a child with rare disease.

**Table 1:** Participant demographics and patient engagement profile

Participant Demographics	
Identifies as Hispanic/Latino	*N* (%)
Yes	7 (64%)
No	4 (36%)
Spanish Fluency Levels	
Fluent	7 (64%)
Advanced	1 (9%)
Intermediate	1 (9%)
No Fluency to Beginner	2 (18%)
Types of Cultural Engagement	
Both	7 (64%)
Only Professional	4 (36%)
Only Personal	0 (0%)
Types of Patient Interactions	
Spanish Speaking and Rare Genetic Disease Patient Interactions	9 (82%)
Only Spanish Speaking Patient Interactions	2 (18%)
Only Rare Genetic Disease Patient Interactions	0 (0%)
Frequency of Interactions with Spanish Speaking Patients	
Daily	6 (55%)
Weekly	4 (36%)
Monthly	1 (9%)
Never	0 (0%)
Frequency of Interactions with Rare Genetic Disease Patients	
Daily	6 (55%)
Weekly	1 (9%)
Monthly	1 (9%)
Never	3 (27%)

**Table 2:** Participant job titles

Participant	Participant job title
M1	Medical Geneticist
M2	Genetic Counselor
M3	Genetics Fellow
M4	Genetic Counselor
M5	Pediatric Optometrist
S1	Hospital Language Program Manager
S2	Rare Disease and Health Equity Specialist
S3	Public Health Language Coordinator
S4^a^	Director for Patient Rare Disease Organization
S5	Volunteer Medical Interpreter
S6	Senior Manager of Hospital International Department

*M* medical professional category, *S* support professional category

^a^ Parent of a child with rare disease

### Overarching codes and themes

**Fig. 1:**
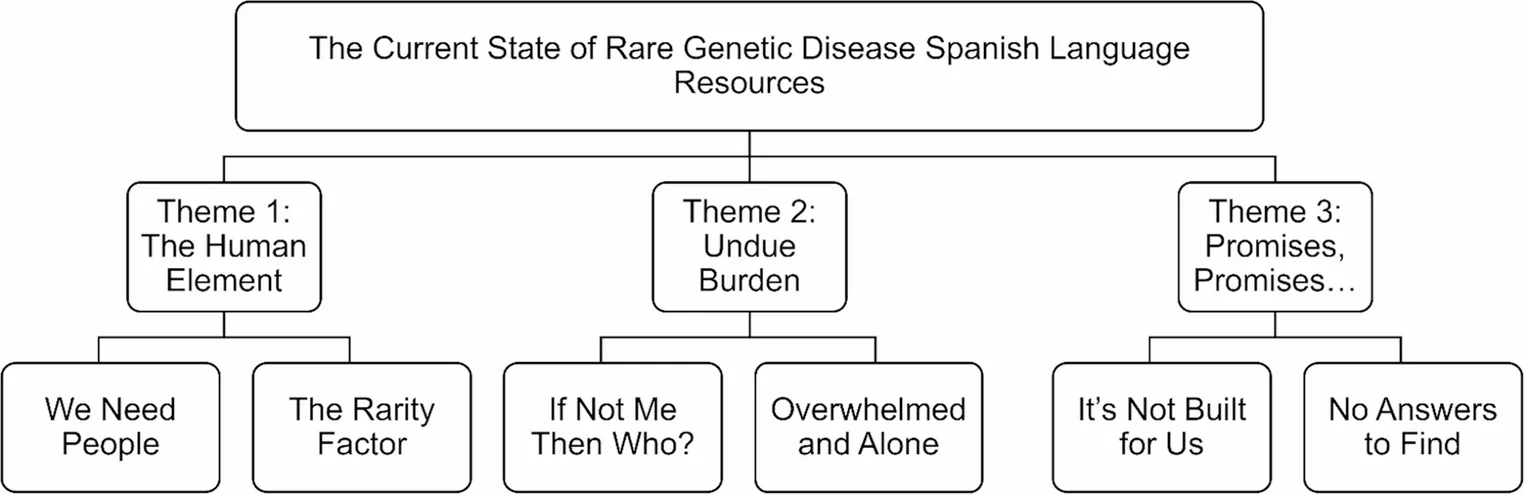
Themes and subthemes flowchart

Reflexive thematic analysis of participant interview data identified three main themes, each with two subthemes (Fig. 1). All participants expressed a lack of availability and overall dissatisfaction with the current state of Spanish language resources for patients with rare genetic disease in the United States. However, the consensus was that currently existing Spanish language resources are generally good quality. All participants also expressed optimism about the growing state of resources for this population moving into the future.

### Theme 1: the human element

The idea of “human resources”, or people and relationships acting as a resource, was repeatedly mentioned as sources of medical information and social support. However, language and cultural barriers prevent patients from accessing these “human resources” for assistance, education, or basic human interaction.

*M2: “There have been several cases too*,* where we find a diagnosis for a patient who is Spanish speaking and there is a support group [for their condition]. Then*,* I talk about how this is available*,* but I couldn’t find a Spanish support group. It’s just always disheartening to see these patients[…] see their faces kind of fall because they don’t have a specific group to reach out to. So*,* that’s a more general experience that I’ve witnessed several times at this point.”*

#### Subtheme 1.1: we need people

A recurring topic was the lack of availability of healthcare staff prevents quality care for Spanish speaking patients with a rare genetic disease. Participants mentioned the pitfalls of shortages of people who speak Spanish throughout the medical system. Participants highlighted the benefit of in-person interpreters over video or phone interpretation services as a resource, especially in emergent or emotionally sensitive situations.

*S6: “We have video interpretation readily available all the time*,* but there are some circumstances in which you need an in-person interpreter. Let’s say that there is a code [hospital emergency or patient needing resuscitation]*,* while you come and get the video interpretation cart*,* and you connect with the interpreter*,* that might be too late. [In comparison] with an English-speaking family*,* [when] there is a code […] someone is able to explain right away what’s happening and why the medical teams are doing what they have to do. That’s not the case for when you don’t speak English*,* if you don’t have the interpreter right there [with immediate access]”.*

Many participants also shared negative impacts of interpretation on patient-provider relationships. They reported the process of interpretation, both in-person and through audiovisual technology, made it difficult to build rapport and connect with patients and families, creating an additional barrier to access “human resources” for patients who speak Spanish.

Many participants also discussed the importance of having cultural knowledgeable staff regardless of role or language spoken. All participants communicated that having access to bicultural and bilingual healthcare staff was important in providing quality care to Spanish speaking patients with rare genetic disease. They argued that if personnel were not familiar with cultural norms, they could not be effective resources for this patient population.

*S1: “[Having] trained personnel is so important because we can have the resources*,* but if the personnel that we have [are] not trained to understand[…] the Latino community and all the differences that they are facing every day*,* we’re still on the same page [lacking proper support][…] The thing is what we need to find is the right people*,* or the people who are trained correctly*,* to understand how the community drives and […] their belief[s] or feelings or any other cultural background issues”.*

One medical professional described the invaluable role that their in-person interpreter played in helping cross cultural barriers and allow them to attend a funeral for a Spanish speaking child.

#### Subtheme 1.2: the rarity factor

Participants frequently mentioned the importance of community outside of the healthcare system as a resource for patients. The rarity of patient conditions can prohibit community building on top of existing geographic and language divides. Participants shared that having a diagnosis often left patients disappointed since finding a diagnosis did not automatically mean finding a community.

*M1: “I had a parent one time tell me ‘It’s nice knowing [gene name]’*,* but at the time there was one case report. She said*,* ‘I thought I would feel so good once we knew the condition*,* but now I just wish this thing had a name’. I think she was looking for an eponym or something that she could Google and search*,* but it can be really hard when you think you found a gene that causes a child’s rare disease but that’s just the start of it. And there’s really not a community around that.”*

Many participants identified patient-run organizations as useful resources in the rare genetic disease space. Medical professional participants highlighted that patient-run organizations or Facebook groups were often the only patient-friendly resources in English on rare genetic disease. Therefore, participants felt that Spanish speaking patients were barred from participating in this community when these resources were not translated.

*M2: “That again is another barrier for these families*,* where they can’t find a support group of families like them who don’t speak English or don’t speak English well. So*,* they may not be comfortable joining those support groups and they’re essentially left without anything at that point.”*

### Theme 2: undue burden

Participants explained that when resources were not available providers or patients were left to fill the gap. Medical professionals shared they felt the need to choose between either taking on the extra work of supplementing resources or providing inferior care to their patients who speak Spanish. Many participants identified that when providers were not able to find accessible resources their patients were left to search for resources themselves.

*M1: “I worry that the patients sometimes feel like they’re being a bother*,* because I’ve had patients before say*,* ‘don’t feel like you have to wait in the interpreter. I’ll get by.’ Or they’ll try to use minimal English*,* or they’ll try to use a family member as an interpreter. And it’s like*,* ‘no*,* no*,* like I wanna do right by you. I’m sorry there’s a wait.’ but I just don’t ever want them to think that they’re wasting time or making things harder for us because it’s their right to have communication in the way that’s best for them.”*

#### Subtheme 2.1: if not me then who?

Many of the medical professionals discussed their frustration and expressed guilt over both the current state of resources and the responsibility of finding resources being placed on patients. Almost all participants shared they take extra time to find or create personalized resources for their Spanish speaking patients. They used either their own Spanish language skills or access to bilingual staff to create resources or hand translate available English resources.

*M2: “I struggle to find these appropriate resources for families who are Spanish preferring. I often just have to go to Google and type in what I’m looking for and ask for Spanish resources or type it in Spanish to see if something comes up. So*,* it can be pretty challenging for me as well. And again*,* that’s really why we decided to just start creating our own resources for these families.”*

Outside of the time needed to create resources, some providers put in an enormous amount of time and effort to make up for health system gaps. For providers who speak Spanish, this could mean becoming extremely involved in all levels of a patient’s care. One medical professional shared that when there was no appropriate Spanish language resource to help navigate the complex healthcare system for their patient the participant became that resource.

*M5: “ [For a patient I referred] I FaceTimed while she was being seen there so that she could understand why she was getting swabbed [for genetic testing]. So*,* it’s a little more challenging than referring an English speaker. Because if I need genetics [for] an English speaker*,* I can say*,* ‘Okay*,* you’ll hear from genetics. They’ll make you an appointment.’ But if I have a Spanish speaker*,* I have to be involved in every part of the process. So*,* that’s a little more time consuming*,* […]you become that person for them[… ]as part of your job.”*

#### Subtheme 2.2: overwhelmed and alone

Multiple participants noticed patients’ feelings of isolation due to the lack of accessible resources. Several participants pointed out the need for additional support during stressful experiences and the lack of proper resources amplifying patients’ feelings of loneliness. One medical provider shared their experience with a Spanish speaking family and their critically ill child.

*M1: “From a medical perspective*,* this child is in [emergent] respiratory distress. You have to move quickly; you have to handle things. I don’t even remember if we were able to have an in-person interpreter there when they came into the emergency room at that time. I’m sure that must have been extremely overwhelming and concerning for them.”*

In this case, the provider thought not having access to a medical interpreter increased the family’s stress and feelings of being alone. Several other providers highlighted that patients and families also face isolation during non-emergent situations. Participants discussed the difficulty of asking basic questions like how to order food during their child’s hospital admission and seeing family members of patients who traveled for care never leave the hospital because they could not easily navigate an unfamiliar city due to a language barrier.

### Theme 3: promises, promises…

According to participants, when Spanish language resources were available they had inconsistent implementation. Having resources advertised as available did not mean they were accessible. Instead, language concordant resources were only available at certain times or for specific situations. One participant mentioned an experience where bilingual resources were advertised on an organization’s website, but upon calling for more details learned the resources were no longer offered. Multiple participants pointed out that websites for rare genetic disease organizations often only have a portion of their pages translated.

*S4: “Some of [the patient support organizations] do have [their website] in Spanish*,* but some of them…it’s only the front page*,* but nothing else. So*,* it’s just saying that you translated one*,* just one*,* page of the website and then everything else in English. That really does nothing*,* right? […] Just checking a box saying that I translated something into Spanish*,* but it’s not really translated.”*

The inconsistent implementation of resources was not just with support organizations, but within hospital systems. One participant’s hospital had translated educational resources available for some conditions, but not all. Two participants worked in different departments within the same hospital system and had conflicting experiences. The participant in hospital leadership stated that resources, like support groups and handouts, should be available for all providers to use. However, the provider from the same hospital explained that in practice these resources were difficult to access.

Another layer of inconsistency was with interpretation quality and availability. Many participants mentioned that when in-person interpreters were not available they had to use audio or video interpreters, which were usually lower quality. One medical professional shared that although their hospital system had in-person interpreters, they could only be used with a subset of patients.

*M4: “I do have access to in-person translators. I have two really wonderful Spanish interpreters that I work with*,* but they are only available to the patients who come through this one specific program. While those are really helpful*,* I can’t use them all the time for all my Spanish speaking patients. That feels unfair and makes it harder to work with some of those families”.*

Participants who did not speak Spanish felt powerless and were unable to judge if the patient was receiving accurate information through interpretation and translated resources. Inaccurate translations were mentioned by many participants, including concerns about the use of machine translation leading to inaccurate or awkward phrasing.

#### Subtheme 3.1: it’s not built for us

Every participant discussed how the American healthcare system was not built to provide resources to fully meet the needs of the Spanish speaking patient population. Participants described innumerable aspects of the healthcare system that cannot accommodate someone who speaks another language.

*S6: “Every touch point of the patient care continuum [needs to be available in Spanish]. When we call them to give them instructions before a procedure[…]*,* when they call the pharmacy to refill prescriptions*,* when we give them the bottles with the medication and the labels are in English. […] [Spanish speaking patients face] challenges every step of the way if there are not proper language resources.”*

This argument was reiterated by several participants who shared their hospital signage and paperwork is only in English. Although hospital discharge paperwork may be interpreted verbally to families before leaving the hospital, there is still a barrier to access because families cannot reference the paperwork once they return home.

Furthermore, several participants mentioned that the lack of Spanish language access in larger systems outside of medicine impact needs associated to a patient’s rare genetic disease. For example, multiple participants brought up the school system and the necessity for medically complex children to have accommodations through individual education plans. They explained that language barriers prevented parents from engaging with teachers and ensuring accommodations were implemented. Participants also mentioned the legal system and cited experiences where language or cultural barriers further complicated the immigration process for families needing to receive rare genetic disease care or treatment in the United States.

*M4: “I had [an experience] where the child had a rare genetic condition and his dad was undocumented and was like actively being deported. One thing that I got asked to do was write a letter in support of having him stay [in the United States]to help take care of this medically complex kid. I wasn’t asked to do anything in another language*,* but that was a very unique situation that I’ve not had to be in for any of my English-speaking patients.”*

Multiple participants mentioned the evolving political landscape in the United States added stress on patients and their ability to receive proper medical care. Participants emphasized that facilitating legal resources is a unique need for this patient population that their English-speaking patients rarely face.

#### Subtheme 3.2: no answers to find

Even when quality resources exist and are available, patients still have difficulties in accessing them. Many participants explained that outside factors, including personal choices and values, impacted whether patients could access available Spanish language resources. Participants shared that many in the Hispanic/Latino community preferred to receive information through audio media or word of mouth rather than physical or electronic print. Additionally, when patients were unable to access computers or technology this eliminated access to educational and social support resources available exclusively online. Several participants mentioned that education levels and health literacy play a large part in whether resources are accessible. One support professional, who also had a child with a rare disease, explained how health literacy was a barrier for them.

*S4: “When my daughter was a diagnosed six years ago ─ even though I spoke some English*,* even I am an educat[ed] person*,* I have a master’s degree ─ it was hard for me to understand because I don’t have a background in science. So*,* it was very hard for me to understand the details of the information I was finding about my daughter’s rare disease.”*

## Discussion

The purpose of this study was to explore the current landscape of Spanish language resources for individuals with rare genetic disease within Alabama. The reported resource needs of the Spanish speaking population were similar to those reported by English speakers (Belzer et al. 2022; Bogart et al. 2022; Bryson and Bogart 2020), with exception of this population’s need for legal and immigration related resources. Overall, participants from this study agreed with previous findings that Spanish language rare genetic disease educational resources that do exist are usually of good quality–refer to Supplemental Material 4 for participant recommendations (Westrate et al. 2020). However, similar to previous literature, participants also expressed dissatisfaction with the quantity and accessibility of Spanish language resources for genetic conditions (Chung et al. 2023; Litzkendorf et al. 2020; Westrate et al. 2020). While there may be additional resources in existence for this population in other Spanish-speaking countries that could give some benefit to patients in the United States, resources from other countries may be specific to that country’s culture, language and dialect, healthcare systems, patient needs, and available resources. Resources from other countries may also be more difficult for providers and patients to identify and use due to logistics such as differing time zones and search engine algorithms (Kliman-Silver et al. 2015). This would leave persisting unmet needs for patients with rare diseases who speak Spanish in the United States. Spanish support and resources from other countries was not purposefully excluded from this research. However, the fact that all but one of our participants practiced exclusively in the United States limits our results to resources available in the United States. Despite this, all participants felt a sense of optimism regarding the future due to recent growth of Spanish-translated resources for those with rare genetic diseases.

The importance of quality “human resources” was notable throughout this study. All participants saw value in using interpreters, but many noted limitations of their use depending on the interpreter’s level of cultural knowledge and interpretation modality. This is supported by previous literature suggesting language and cultural differences negatively impact patient-provider communication even when using in-person interpreters (Welty et al. 2012). This study’s expert stakeholders preferred access to bilingual healthcare staff to remove any negative impacts of third-party interpreters. However, only employing bilingual staff in a healthcare system is not a reasonable solution, especially when very few genetics professionals are bilingual (Matalon et al. 2023). Furthermore, being bilingual does not guarantee cultural understanding as the cultural background within the Spanish speaking community is diverse. Therefore, some participants suggested cultural competency and cultural intelligence training for all hospital employees to help bridge this cultural gap. Cultural competency, sometimes called cultural humility, training has been shown to reduce challenges and barriers facing patients and increase provider understanding of the disparities facing minority population groups (Vella et al. 2022). Therefore, full integration of cultural training could help close this gap.

Outside of medical providers, community and social support were identified as important resources for rare genetic disease patients, mirroring previous findings in the literature (Bogart et al. 2022). However, patients who speak Spanish are often prevented from joining such groups because they are usually only available in English (Tones et al. 2023). The participants also shared that disease rarity often impacts the quantity of available resources. This is similar to previous reports from English speaking patients who seek social support from organizations adjacent to their child’s actual diagnosis, for example broader rare disease or children with disabilities groups, because there are no disease specific support groups (Baumbusch et al. 2019).

Participants believed that providers and patients are forced into filling the Spanish language resource gap themselves. Providers recounted extra time and effort needed to create personalized Spanish language resources, and the burden placed on patients when providers do not put in this extra effort. This is on top of already established health disparities for Spanish speaking or otherwise marginalized population groups (Radley et al. 2024; Matalon et al. 2023). This is especially concerning considering it has been established that individuals with rare genetic disease have lower quality of life and higher levels of mental health conditions compared to the general population (Bogart et al. 2022).

Although participants thought existing Spanish language resources for genetic rare disease were generally high-quality, a reported problem was inconsistent implementation of and access to these resources. Participants from this study discussed frequent experiences with websites only partially translated or incorrectly and inaccurately translated with machine translation, echoing previous findings (Westrate et al. 2020). Although machine translation or use of artificial intelligence (AI) not translate resources may help with resource accessibility, AI technology it not yet a viable solution for effective closing of the resource gap. Participants in this study shared similar concerns to those in previous studies regarding patients receiving misinformation about rare genetic diseases due to inaccurate Spanish language resources (Pogue et al. 2018). Collecting reliable resources in a nationwide repository could reduce the potential for misinformation while improving resource access and utilization for healthcare providers and patients.

Although the focus of this study was rare genetic disease resources, a recurring theme from the participants were general challenges within the United States and the impacts of navigating the healthcare, school, and legal systems with a genetic rare disease while speaking Spanish. Although interpreters may be available for patient appointments, additional translations outside of scheduled appointments are often overlooked. Things like medical center signage, pharmaceutical labels, and discharge paperwork were rarely translated. Additionally, many patients and families are limited in the resources they can use due to lack of access to technology and education and health literacy levels. Although not specifically asked about, the lack of translation within the medical system combined with other access barriers may help explain why Latino families are less likely to participate in support programming or educational interventions (Cohen et al. 2014).

Although this study was intended to be through a lens of Alabama resource access, being in Alabama was not an independent inclusion criterion given the niche professional experiences of the target participants. Although no Alabama specific needs or challenges were identified, there may be state specific perspectives not captured by this research. Potential differences in resource availability and use between states or regions with larger and/or more established Spanish-speaking populations should be explored further. This study also did not focus on patient perspectives about Spanish language resources, although one participant happened to be both a support professional and a parent to a child with a rare genetic disease. Additionally, interviews were completed in English and individuals who only speak Spanish were excluded. Although including participants who only speak Spanish would have added depth to this study, logistical limitations and the qualitative nature of this study were a barrier to their inclusion. The process of interpretation and translation could have altered the meaning of participant responses which would have negatively impacted the analysis and subjective perception of participant quotes. Most participants from this study work with pediatric patients with rare genetic diseases, so experiences for adult patients may not have been fully captured. Future research including these additional perspectives would be insightful to further guide Spanish language resource curation for patients with rare genetic disease. Another potential area of research is exploring these issues through the lens of other languages, especially minority languages. Spanish is the second most common language spoken in the United States, therefore improvements in Spanish-language resources have the potential to improve healthcare experiences for the largest number of patients at this time (Dietrich 2022). However, all patients deserve access to healthcare and patient education, no matter how common their language is spoken in the United States. Replicating this type of research with a broader language focus would be a great step in improving resource access for these patients.

## Conclusions

Overall, this study adds to the body of literature establishing that a lack of Spanish language resources adds burden to both providers and patients with rare genetic diseases. Existing Spanish resources are often thought to be high quality; however, their availability is limited. A list of existing Spanish language resources that participants reported positive experiences with are listed in Supplemental Material 4. Based on this study’s findings, organizations should focus on expanding translation and interpretation access and standardizing the implementation of current resources. However, additional expansion of Spanish language resources could include creating audio-only versions of existing resources to better engage the Spanish-speaking community and reduce the impact of literacy level as a barrier.

## Supplementary Information

Below is the link to the electronic supplementary material.


Supplementary Material 1


## Data Availability

The data that support the findings of this study are not openly available due to reasons of sensitivity and are available from the corresponding author upon reasonable request.

## Declarations

All authors provided final approval for the version to be published and agree to be accountable for all aspects of the work.
