# Supplementary material for: The current state of Spanish language resources for patients in Alabama with rare genetic disease: qualitative expert stakeholder interviews
Source: J Community Genet. 2026 Jul 31;17(4):93. doi: 10.1007/s12687-026-00928-1 (PMC13424011; doi:10.1007/s12687-026-00928-1)
Supplement: Supplementary file 1 — Supplementary Material 1 [file 12687_2026_928_MOESM1_ESM.docx]

# **Supplemental Material**

The Current State of Spanish Language Resources for Patients with Rare Genetic Disease: Qualitative Expert Stakeholder Interviews

Journal of Community Genetics

Spencer Elizabeth Favor, MS, CGC

Alicia Gomes, MS, CGC

Carlos Javier Torres, MS

Katie Church, MS, CGC

Corresponding author**:** Alicia Gomes, MS, CGC, University of Alabama at Birmingham, agomes@uab.edu

## **Supplemental Material 1: Intake Questionnaire**

**Section 1: Inclusion Criteria**

1. I am English-speaking and would be able to complete the interview in English.
   1. Yes
   2. No
2. In my professional role, I engage with and/or my work impacts the Spanish-speaking population.
   1. Yes
   2. No
3. If yes, how often?
   1. Daily
   2. Weekly
   3. Once per month
   4. Less than once per month
4. In my professional role, I engage with and/or my work impacts populations in Alabama.
   1. Yes
   2. No
5. If yes, how often?
   1. Daily
   2. Weekly
   3. Once per month
   4. Less than once per month
6. In my professional role, I engage with and/or my work impacts populations with rare genetic diseases.
   1. Yes
   2. No
7. If yes, how often?
   1. Daily
   2. Weekly
   3. Once per month
   4. Less than once per month
8. Please select if your professional role or work falls into any of the following categories
   1. Social assistance
   2. Community assistance
   3. Financial assistance
   4. Medical care
   5. Resource creator
   6. Other (please specify):
9. Please provide your Professional job title here:

**Section 2: Contact information**

1. Name:
2. Email address:
3. Phone number:
4. Communication Preference
   1. Email
   2. Phone

**Section 3: Snowball Recruitment**

1. If you know of anyone else may be a good candidate for participation in this study, please provide their information.
   1. Name:
   2. Professional job title:
   3. Email address:

## **Supplemental Materials 2: Interview Guide (Combined Medical and Support)**

**Section 1: Participant Characteristics and Background**

1. What languages do you speak and how would you describe your proficiency in them?
   1. What is your experience with the Spanish language?
2. How would you describe your cultural background?
   1. Do you identify as Hispanic and/or Latino?
   2. How would you describe your cultural engagement with the Hispanic/Latino community?
3. If you had to estimate, what percentage of the [patients/clients] you engage with professionally are individuals who are English-speaking?
4. What percentage are Spanish speaking?
5. If you had to estimate, what percentage of the [patients/clients] you engage with professionally has or is at risk for a rare genetic disease?

**Section 2: Experience with Spanish-Speaking Patients**

1. Based on your experience, what are the biggest differences in challenges between [patients/clients] who are not English-speaking and those who are?
   1. What challenges do your Spanish-speaking [patients/clients] tell you about?
      1. Are these different from the challenges shared by those who are English-speaking?
   2. When thinking more specifically about Spanish-speaking [patients/clients] who also have rare genetic disease, what challenges do they tell you about?

**Section 3: Current Resource Use Practices**

1. What kind of handouts, websites, support groups and resources do you use and/or recommend the most for your [patients/clients] in general?
   1. How often do you use these resources?
   2. What kind of resources do your patients/clients request the most?
   3. Are there resources to fill these requests?
   4. When resources are not available, what do you do?
2. If you had to identify a gap in the currently available resources, where do you think the biggest gap is?
   1. When thinking about the resources you currently use with patients, how would you compare their use with Spanish-speaking [patients/clients] compared to English-speaking patients/clients?
      1. What have your [patients/clients] shared with you about resource access and availability?
      2. Are the resources you suggest for your Spanish and English-speaking [patients/clients] different?
   2. How would you describe the quality and current state of resources for Spanish-speaking [patients/clients]?
3. Have you noticed any differences in availability of resources between Spanish speaking patients and English-speaking [patients/clients]?
   1. [*Support professional only*] What differences are there based on where the resource comes from?
   2. [*Support professional only*]What barriers exist for clients accessing these resources?
      1. Are these barriers different for Spanish vs English-speaking [patients/clients]?
   3. Have you noticed a difference between Spanish and English-speaking [patients/clients] being able to find resources themselves?
   4. Are you confident in [patients’/clients’] ability to find resources on their own?
4. How would you describe the quality and current state of resources for [patients/clients] with a rare genetic disease?
5. What about resource availability for those who are Spanish-speaking AND have a rare genetic disease?
6. Can you tell me about any specific experiences that you have or that made an impact on you?

**Section 4: Moving Forward**

1. If you could design your ideal resources for Spanish-speaking [patients/clients] with rare genetic disease, what would it look like?
   1. What information would it include?
   2. What format would this be in?
   3. How would this be used
   4. What other aspects would you consider?
2. In your opinion, do you see the landscape of resources for Spanish-speaking rare genetic disease [patients/clients] evolving in the future?

**Section 5: Conclusion**

1. What else would you like to share with us about resources for Spanish speaking [patients/clients]?

## **Supplemental Material 3: Standards for Reporting Qualitative Research (SRQR) Checklist (O’Brien et al., 2014)**

| **Title and abstract** | **Line no(s).** |
| --- | --- |

| **Title** - Concise description of the nature and topic of the study Identifying the study as qualitative or indicating the approach (e.g., ethnography, grounded theory) or data collection methods (e.g., interview, focus group) is recommended | 1-2 |  |
| --- | --- | --- |
| **Abstract** - Summary of key elements of the study using the abstract format of the intended publication; typically includes background, purpose, methods, results, and conclusions | 16-36 |  |
|  |  |  |
| **Introduction** |  | |
| **Problem formulation** - Description and significance of the problem/phenomenon studied; review of relevant theory and empirical work; problem statement | 38-98 |  |
| **Purpose or research questio**n - Purpose of the study and specific objectives or questions | 98-102 |  |
|  |  |  |
| **Methods** |  | |
| **Qualitative approach and research paradigm** - Qualitative approach (e.g., ethnography, grounded theory, case study, phenomenology, narrative research) and guiding theory if appropriate; identifying the research paradigm (e.g., postpositivist, constructivist/ interpretivist) is also recommended; rationale** | 138-155 |  |
| **Researcher characteristics and reflexivity** – Researchers’ characteristics that may influence the research, including personal attributes, qualifications/experience, relationship with participants, assumptions, and/or presuppositions; potential or actual interaction between researchers’ characteristics and the research questions, approach, methods, results, and/or transferability | 141-146 |  |
| **Context** – Setting/site and salient contextual factors; rationale** | 105-114 |  |
| **Sampling strategy** - How and why research participants, documents, or events were selected; criteria for deciding when no further sampling was necessary (e.g., sampling saturation); rationale** | 116-122 |  |
| **Ethical issues pertaining to human subjects** - Documentation of approval by an appropriate ethics review board and participant consent, or explanation for lack thereof; other confidentiality and data security issues | 105-106, 119-120, 133-134 |  |
| **Data collection methods** - Types of data collected; details of data collection procedures including (as appropriate) start and stop dates of data collection and analysis, iterative process, triangulation of sources/methods, and modification of procedures in response to evolving study findings; rationale** | 126-132 |  |
| **Data collection instruments and technologies** - Description of instruments (e.g., interview guides, questionnaires) and devices (e.g., audio recorders) used for data collection; if/how the instrument(s) changed over the course of the study | 132-134 |  |
| **Units of study** - Number and relevant characteristics of participants, documents, or events included in the study; level of participation (could be reported in results) | 158-175 |  |
| **Data processing** - Methods for processing data prior to and during analysis, including transcription, data entry, data management and security, verification of data integrity, data coding, and anonymization/de-identification of excerpts | 116-122, 133-134 |  |
| **Data analysis** - Process by which inferences, themes, etc., were identified and developed, including the researchers involved in data analysis; usually references a specific paradigm or approach; rationale** | 138-155 |  |
| **Techniques to enhance trustworthiness** - Techniques to enhance trustworthiness and credibility of data analysis (e.g., member checking, audit trail, triangulation); rationale** | 130-131, 147-155 |  |
|  |  |  |
| **Results/findings** |  | |
| **Synthesis and interpretation** - Main findings (e.g., interpretations, inferences, and themes); might include development of a theory or model, or integration with prior research or theory | 158-400 |  |
| **Links to empirical data** - Evidence (e.g., quotes, field notes, text excerpts, photographs) to substantiate analytic findings | 191-195, 204-210, 222-227. 238-243, 251-254, 263-268, 277-281, 289-294, 303-306, 323-327, 341-344, 356-360, 375-379, 397-400 |  |
|  |  |  |
| **Discussion** |  | |
| **Integration with prior work, implications, transferability, and contribution(s) to the field -** Short summary of main findings; explanation of how findings and conclusions connect to, support, elaborate on, or challenge conclusions of earlier scholarship; discussion of scope of application/generalizability; identification of unique contribution(s) to scholarship in a discipline or field | 403-460 |  |
| **Limitations** - Trustworthiness and limitations of findings | 461-475 |  |
|  |  |  |
| **Other** |  | |
| **Conflicts of interest** - Potential sources of influence or perceived influence on study conduct and conclusions; how these were managed | 490 |  |
| **Funding** - Sources of funding and other support; role of funders in data collection, interpretation, and reporting | 496-497 |  |

## **Supplemental Material 4: Spanish Language Resources Discussed by Participants**

This is a list of Spanish language resources that participants described having positive experiences with. This list is not an endorsement of these resources, the resources on this list have not been formally vetted or evaluated by the authors.

| **Category** | **Organization Name** | **Website** |
| --- | --- | --- |
| **General Rare Disease** | Syndromes Without a Name (SWAN) | https://swanusa.org/ |
|  | Orphanet | https://www.orpha.net/ |
|  | National Organization for Rare Diseases (NORD) | https://rarediseases.org/ |
| **Condition or Body System Specific** | The American Ophthalmology Society | https://www.aao.org/ |
|  | Unique | https://rarechromo.org/ |
|  | Lucky Finn Foundation | https://luckyfinproject.org/ |
| **General Health** | Medline Plus | https://medlineplus.gov/spanish/ |
|  | Healthwise (now WebMD Ignite) | https://webmdignite.com/ |
| **Alabama Specific** | InterpretES Medical Student-led Community Service | No website, available through the Office of Service Learning at the University of Alabama at Birmingham |

# **Supplemental Materials References**

O’Brien, B. C., Harris, I. B., Beckman, T. J., Reed, D. A., & Cook, D. A. (2014). Standards for Reporting Qualitative Research: A Synthesis of Recommendations. *Academic Medicine*, *89*(9).
